# Empagliflozin Repurposing for Lafora Disease: A Pilot Clinical Trial and Preclinical Investigation of Novel Therapeutic Targets

**DOI:** 10.3390/mps8030048

**Published:** 2025-05-06

**Authors:** Giuseppe d’Orsi, Antonella Liantonio, Paola Imbrici, Nicola Gambacorta, Giorgia Dinoi, Cosimo Damiano Altomare, DEFEAT-LD Study Group, Massimo Carella

**Affiliations:** 1 Neurology Unit, Fondazione IRCCS Casa Sollievo della Sofferenza, 71013 San Giovanni Rotondo, Italy; 2 Department of Pharmacy—Drug Sciences, University of Bari “Aldo Moro”, 70121 Bari, Italy; antonella.liantonio@uniba.it (A.L.); paola.imbrici@uniba.it (P.I.); nicola.gambacorta1@uniba.it (N.G.); giorgia.dinoi@uniba.it (G.D.); cosimodamiano.altomare@uniba.it (C.D.A.); 3 Division of Medical Genetics, Fondazione IRCCS Casa Sollievo della Sofferenza, 71013 San Giovanni Rotondo, Italy; m.carella@operapadrepio.it

**Keywords:** Lafora disease, empagliflozin, glycogen, molecular docking

## Abstract

Background: Lafora disease (LD) is an ultra-rare and fatal neurodegenerative disorder with limited therapeutic options. Current treatments primarily address symptoms, with modest efficacy in halting disease progression, thus highlighting the urgent need for novel therapeutic approaches. Gene therapy, antisense oligonucleotides, and recombinant enzymes have recently been, and still are, under investigation. Drug repurposing may offer a promising approach to identify new, possibly effective, therapies. Methods: This study aims to investigate the conditions for repurposing empagliflozin, an SGLT2 (sodium/glucose cotransporter-2) inhibitor, as a potential treatment for LD and to establish a clinical protocol. Clinical phase: This 12-month prospective observational study will assess the safety and clinical efficacy of empagliflozin in two patients with early to intermediate LD stage. The primary endpoints will include changes in the severity of epilepsy and cognitive function, while the secondary endpoints will assess motor function, global function, and autonomy. Multiple clinical and instrumental evaluations (including MRI and PET with ^18^F-fluorodeoxyglucose) will be performed before and during treatment. Safety monitoring will include regular clinical assessments and reports of adverse events. Preclinical phase: In silico studies (using both molecular docking calculations and reverse ligand-based screening) and in vitro cell-based assays will allow us to investigate the effects of empagliflozin (and other gliflozins) on some key targets likely implicated in LD pathogenesis, such as GLUT1, GLUT3, glycogen synthase (hGYS), and glycogen phosphorylase (GP), as suggested in the literature and digital platforms for in silico target fishing. Results: The expected outcome of this study is twofold, i.e., (i) assessing the safety and tolerability of empagliflozin in LD patients and (ii) gathering preliminary data on its potential efficacy in improving clinical and neurologic features. Additionally, the in silico and in vitro studies may provide new insights into the mechanisms through which empagliflozin may exert its therapeutic effects in LD. Conclusion: The findings of this study are expected to provide evidence in support of the repurposing of empagliflozin for the treatment of LD.

## 1. Introduction

Lafora disease (LD, OMIM 254780, ORPHA 501) is an ultra-rare, genetically inherited (autosomal recessive), and fatal form of progressive myoclonic epilepsy [1]. LD is characterized by rapid neurological deterioration linked to the progressive appearance of insoluble glycogen-like inclusions (polyglucosan), known as Lafora bodies (LBs), in the brain and peripheral tissues [2]. Mutations in two genes, *epm2A* and *epm2B/NHLRC1*, located on chromosome 6 and encoding laforin and malin, two proteins involved in glycogen metabolism, are responsible for the gradual accumulation of LBs and subsequent neurodegeneration [3].

The prevalence of LD is estimated at about 300 cases worldwide, but this number is likely underestimated due to diagnosis uncertainty. LD is relatively more frequent in Mediterranean countries, particularly in Italy. According to data from the Italian Lafora Association (AILA), there are currently 25–30 patients in Italy, with about one-third located in Apulia, a region in southeastern Italy [4]. Furthermore, there is a higher prevalence of mutations in the *epm2B* gene in Italian patients, suggesting a potential founder effect for certain pathogenic variants. In the Apulia region, specifically, at least two clusters of patients with the same type of mutation have been identified [4].

LD typically appears in individuals between the ages of ten and fifteen, who initially have a normal neurodevelopment, followed by generalized tonic–clonic seizures, visual focal seizures, and myoclonus. Diagnosis is often delayed, and distinguishing LD from juvenile myoclonic epilepsy can be difficult [4]. As the disease progresses, epilepsy becomes refractory and is associated with continuous myoclonus and progressive neurological deterioration with dementia, ataxia, and photosensitivity. Gradually, patients enter a vegetative state, with death occurring within eleven years of symptom onset [5] due to neurological problems such as refractory or super-refractory status epilepticus or medical complications like aspiration pneumonia [6].

The optimal therapy for LD should be based on early intervention to prevent rapid neurological decline and to inhibit the formation of LBs, which underlie neurodegeneration. Currently, the only available therapeutic approaches in LD are symptomatic and ineffective at halting disease progression, and they are usually performed at a late stage of the disease. These treatments mainly involve anti-seizure medications, with metformin and the ketogenic diet being additional options. The antidiabetic drug metformin received orphan drug designation for LD in 2016 by EMA (EU/3/16/1803) and in 2017 by FDA (12/14/2017), based on the efficacy found in animal models of LD, which have shown a mechanism of action partly dependent on the indirect activation of AMP-activated protein kinase (AMPK). However, the effectiveness of metformin in treating LD patients remains unclear, possibly due to its administration at a medium-advanced stage of the disease. Early treatment might likely provide better results in slowing disease progression [7,8,9]. The ketogenic diet, which promotes the production of neuroprotective ketone bodies and limits glucose intake, may reduce glycogen synthesis, potentially decreasing polyglucosan accumulation and slowing down the disease progression. Nevertheless, efficacy data obtained from mouse models have not been confirmed in humans, as the ketogenic diet has been tested in patients with advanced LD [10,11].

Innovative therapeutic approaches are currently being evaluated in animal models and human trials and are based on the reduction in brain glycogen synthesis (antisense oligonucleotides), replacement of the non-functional gene (gene therapy), improvements in proteostasis, and neuroinflammation [12,13,14,15]. Recombinant enzymes capable of degrading glycogen and promoting the degradation of LBs are also being investigated [16]. VAL-041 (Valerion Therapeutics) is an antibody–enzyme fusion protein that combines α-amylase (an enzyme involved in pancreatic polyglucan degradation) with an antibody fragment (Fab) to enhance penetration into brain cells. This fusion protein was tested successfully in the *epm2a*-/- mouse. Some Italian LD patients are being treated with the drug rhGAA (recombinant human α-glucosidase, Myozyme^®^), a drug approved by the FDA for Pompe disease, to help degrade lysosomal glycogen. A clinical trial is also underway in Italy with VAL-1221 (Valerion Therapeutics and Parasail), another antibody-enzyme fusion protein based on rhGAA and Fab (EudraCT number 2023-000185-34; Drug discovEry and repurposing to Find a trEAtmenT for Lafora Disease—DEFEAT-LD—PNRR-MR1-2022-12376430). Despite their potential, these innovative therapies face significant challenges in terms of costs and treatment timeline, which are especially critical for a rapidly progressing and fatal disease like LD.

Drug repurposing, a strategy aimed at identifying existing drugs approved for other indications and testing them for new therapeutic uses, may also offer a solution for rare diseases [17]. This approach is expected to reduce both the time and cost of drug development, as repurposed drugs are already approved for use and can be directly tested in preclinical and clinical studies for new pathological conditions, such as rapidly progressing diseases like LD.

**Figure 1:**
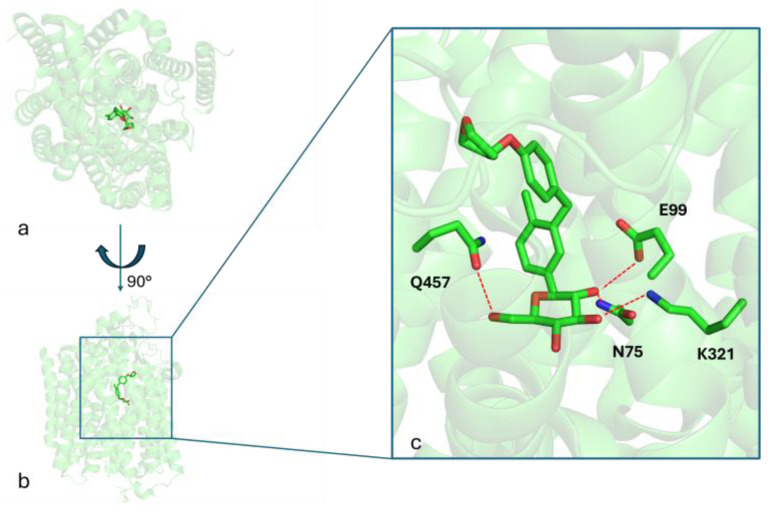
Panels (**a**,**b**) present two distinct orientations of the SGLT2 crystal structure in complex with empagliflozin, highlighting its overall binding conformation. Panel (**c**) provides a detailed close-up view of the empagliflozin binding site, emphasizing key interacting residues involved in ligand binding. Red dashed lines indicate hydrogen bonds formed between amino acid side chains and the ligand.

The aim of this study is to investigate the effects of empagliflozin, a sodium-glucose cotransporter 2 (SGLT2) inhibitor belonging to the gliflozin class, for the treatment of LD. Figure 1 shows the binding mode of empagliflozin into the binding site of the SGLT2 protein (PDB_CODE: 7VSI [18]), as revealed by the crystallography of the ligand–protein complex. Three key hydrogen bonds between the HB-donors 2-OH, 3-OH, and 6-OH of the glucose moiety with the respective HB-acceptors in the side chains of E99, K321, and Q457, along with hydrophobic interactions, stabilize the ligand–receptor complex.

Gliflozins promote renal glucose excretion and exhibit pleiotropic effects. They are indeed approved for treating type 2 diabetes, heart failure, and chronic kidney disease. The potential use of gliflozins in LD is supported by the literature’s data [19]. Several studies have demonstrated the neuroprotective effects of gliflozins in animal models of Alzheimer’s disease and cerebral ischemia through various mechanisms [19,20]. Interestingly, a body of experimental evidence supports the repositioning of empagliflozin for the treatment of glycogen storage disorder (GSD). Empagliflozin has been successfully repurposed in both paediatric and adult patients with GSD type Ib to treat neutropenia and neutrophil dysfunction caused by the accumulation of 1,5-anhydroglucitol-6-phosphate. It has also shown positive effects in improving bowel inflammation in some patients, leading to the amelioration of the quality of life for both patients and caregivers, with good tolerability [21,22,23,24,25,26,27,28]. In addition, a clinical trial testing the effects of empagliflozin in epilepsy is ongoing (NCT05512130). Furthermore, a recent preclinical study using an animal model of LD demonstrated the translational potential of gliflozins in LD patients [29]. Specifically, gliflozins, including empagliflozin, have shown beneficial effects on neuronal hyperexcitability and impaired locomotor activities, key pathological features of LD.

Here, we present the study protocol for administering empagliflozin to LD patients, along with in silico and experimental in vitro approaches adopted to verify its potential in the treatment of LD. The results of this study may provide critical evidence in support of the repurposing of empagliflozin for the treatment of LD.

## 2. Experimental Design

### 2.1. Study Design and Setting

**Objective 1:** The first objective is to evaluate the safety and tolerability of empagliflozin in patients with genetically diagnosed LD in the early to intermediate stages over a 6-month treatment period. Efficacy will be assessed by monitoring changes in the frequency and intensity of epileptic seizures; variations in the background activity and frequency of epileptiform abnormalities on an electroencephalogram (EEG); stabilization of cognitive, behavioral, and motor outcomes, as well as neuro-radiological and metabolic biomarkers (including high-field brain MRI—3 Tesla, brain PET with ^18^F-fluorodeoxyglucose—FDG).

**Objective 2:** The second objective is to investigate the effect of empagliflozin (and SGLT2 inhibitors in general) on targets involved in glucose metabolism and the pathogenesis of LD, aiming to uncover new mechanisms of action and to further support the repurposing of gliflozins in the treatment of LD. Specifically, considering the dysfunctional cellular pathways related to the formation of LBs, two brain glucose transporters (GLUT1 and GLUT3) and glycogen synthase (hGYS) will be explored [30,31]. The interaction with these targets will be evaluated through molecular docking studies, while the ability of gliflozins to reduce intracellular concentrations of glucose and glycogen will be evaluated through in vitro studies using various cell lines.

### 2.2. Eligibility Criteria

A single-center, observational, controlled pilot study will be conducted at the Neurology Unit of IRCCS Casa Sollievo della Sofferenza Hospital in San Giovanni Rotondo (Foggia, Italy). The stage of disease progression will be assessed using a disability scale that considers cognitive and motor functions, activities of daily living, and social interaction [32,33]. The stages are defined as follows: 1—mild cognitive and motor deterioration: preserved activities of daily living and social interaction; 2—moderate cognitive deterioration: limitations in motor activities and social interactions; 3—severe cognitive and motor deterioration: need for assistance with walking and activities of daily living and poor social interaction; 4—wheelchair-bound or bedridden patients: no significant activities of daily living and social interaction. Patients will be enrolled based on the following criteria:

Inclusion Criteria:

- Documented genetic diagnosis of LD (pathogenic mutations in the EPM2A or EPM2B genes);
- Age: 10–22 years;
- Early–intermediate stage of disease (1–2 progression scale) [32,33];
- Patient able to understand and complete the protocol (even with the help of a caregiver over 18 years of age) and perform neuropsychological tests.

Exclusion Criteria:

- Undocumented genetic diagnosis of LD;
- Advanced stage of disease (3–4 progression scale) [32];
- Participation in other ongoing therapeutic treatments for LD;
- Pregnancy;
- Allergy or known hypersensitivity to empagliflozin;
- Other conditions, at the discretion of the investigator, that could interfere with participation or completion of the study.

### 2.3. Discontinuation of Interventions

The investigator will terminate the study if any of the following conditions occur: the emergence of worsening complications; the development of a condition potentially linked to the research; pregnancy; an allergic reaction to the test drug; severe kidney or liver dysfunction; difficulty in continuing the medication; or if the participant is deemed ineligible to continue taking the drug for any other reasons.

### 2.4. Sample Size

This pilot study aims to evaluate the safety and efficacy of empagliflozin initially in two patients with genetically diagnosed LD in the early/intermediate stage of disease. The sample size of two patients was chosen based on the feasibility of recruiting patients with these specific characteristics and the exploratory nature of this pilot study.

### 2.5. Data Collection and Management

Data will be entered into an electronic database. The following baseline data will be collected: informed consent; inclusion/exclusion criteria; demographic data; comprehensive medical and neurological history; detailed history of epileptic seizures, including frequency of generalized tonic–clonic seizures in the last six months (based on previous clinical documentation and/or a seizure diary); and medication history.

All information of the study protocol and the results obtained will be collected by the investigator during the study period and stored at IRCCS Casa Sollievo della Sofferenza Hospital in San Giovanni Rotondo (Foggia, Italy).

### 2.6. Statistical Methods

Descriptive statistics will be used to summarize the data. For continuous variables, measures such as mean, standard deviation, minimum, maximum, and 95% confidence intervals will be calculated. For categorical variables, the frequency and percentage will be reported. Changes from the baseline to follow-up time points will be assessed.

The in silico and in vitro studies proposed here are complementary and will be conducted in parallel with the in vivo human study. The aim of these studies is to gain insight into the additional mechanisms of action of empagliflozin that may be relevant for LD.

### 2.7. In Silico Studies on Target Candidates

Molecular docking calculations will be performed to assess whether empagliflozin can interact with additional targets involved in glucose metabolism and relevant for LD, such as glycogen synthase (hGYS1) and glucose transporters (GLUT1 and GLUT3 isoforms), using Grid-based Ligand Docking with Energetics (GLIDE). To explore differences within the gliflozin class, parallel studies will also be conducted with dapagliflozin and canagliflozin. For each gliflozin, the docking score and ligand efficiency values will be calculated and compared to the respective reference ligand, taken as a positive control of each target protein considered.

As additional investigations, queries in a drug discovery web platform developed by some of us, namely PLATO (the acronym stands for Polypharmacology pLATform prediction), which allows the reverse ligand-based screening of small molecules [34] to be carried out, may allow further investigation into the putative gliflozins’ protein targets that are possibly involved in LB formation and neurodegeneration. Among the priority targets of gliflozins identified by PLATO, there is glycogen phosphorylase (GP), which is implicated in the formation of Lafora bodies and subsequent neurodegeneration.

### 2.8. Validation in Cell Lines

**Figure 2:**
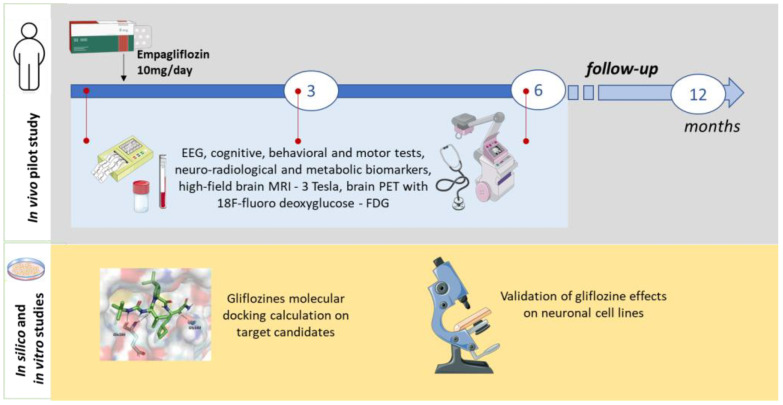
Scheme of the study.

In vitro studies will be conducted to validate the in silico predictions, using biochemical and immunohistochemical assays to quantify glucose and glycogen levels in neuronal and HEK293 cell lines (see Figure 2).

## 3. Procedure

### 3.1. Recruitment

Informed written consent will be obtained from all patients or their legal representatives before inclusion in the study, in line with institutional and national ethical guidelines. Participant will be made aware that their involvement is voluntary and that they can withdraw from the study at any time.

### 3.2. Intervention Description

The drug that we chose to use is empagliflozin. The potential benefit of gliflozins in LD is supported by the following evidence [19,29,35,36,37,38,39]: (1) a relationship is established between altered brain metabolism and the onset of epileptic seizures; (2) the SGLT2 protein is expressed in the brain and its inhibition may reduce both glucose and sodium loads, which may decrease neuronal excitability; (3) gliflozins produce metabolic effects similar to those of the ketogenic diet; (4) ongoing clinical studies are testing empagliflozin in the treatment of epilepsy (https://clinicaltrials.gov/, accessed on 3 May 2025); (5) the neuroprotective effects of gliflozins have been reported in animal models of Alzheimer’s disease and stroke; (6) gliflozins inhibit glycogen accumulation in the kidney in mouse models of glycogen storage disease type 1b and type XI; (7) gliflozins ameliorate phenotypes in LD zebrafish models.

The pharmacokinetic profile of empagliflozin is well documented and has been specifically described in the context of young patients [40,41,42].

Empagliflozin will be orally administered once daily at a dose of 10 mg for 6 months in two patients with LD in the initial or intermediate stage (10–22 years). The proposed treatment duration is based on the pilot nature of the study, as well as on the previous use of empagliflozin in studies involving the treatment of other rare glycogen storage diseases, such as GSD type 1 [23,43].

The goal is to assess its efficacy and tolerability during the presumed phases of initial degeneration and LB accumulation, with the aim of slowing down disease progression.

### 3.3. Participant Timeline

The overall duration of the project is 16 months, with a screening period (4 months), an open-label treatment phase (6 months), and a follow-up period (6 months). Patients will undergo a clinical evaluation at baseline (week 1, before the administration of empagliflozin 10 mg/day), at the intermediate time point (three months), and at the end of treatment (six months). Sub-analyses will also compare seizure rates at intermediate time points. The progression of other LD symptoms will be evaluated at 6 months and compared to the baseline. Changes in FDG-PET and MRI findings will be categorized as stable, improved, or worsened.

### 3.4. Outcomes

The measurement, at 3 and 6 months, of safety and clinical outcomes will be compared qualitatively with prognosis data available in the literature.

The following parameters will be determined: vital signs; weight and height; general and neurological examination; seizure diary; adverse event assessment; ECG; urine and serum pregnancy tests; laboratory tests (hematology, chemistry, and urinalysis); video-EEG/polygraphy; and high-field brain MRI (3 Tesla) and brain PET with ^18^F-fluorodeoxyglucose (at baseline and 6 and 12 months of treatment). Neuropsychological assessments will include the Montreal Cognitive Assessment (MoCA), Leiter Performance Scale 3, Visual Motor Integration test, children’s color trail test, NEPSY-II inhibition subtest, phonemic and semantic verbal fluency test, and Weiss working memory and vocabulary subtests. Motor function assessments will include the 4-stage balance test, timed up and go, 6 min walk test, 10 m walk test, 9-hole peg test, Scale for the Assessment of Ataxia (SARA), and Scale for the Assessment of Myoclonus (UMRS). Assessments of global function and autonomy will include the Lafora Disease Performance Scale (LDS), Lafora Epilepsy Severity Scale (LESS), Activities of Daily Living (ADL) and Instrumental Activities of Daily Living (IADL) scales, Barthel Index, Vineland-II Adaptive Behavior Scales, and Clinical Global Impression scales (CGI-I and PGI-CaGI-C). All assessments will be performed by the same investigator and video-recorded (with informed consent).

The following endpoints for the safety and efficacy of empagliflozin will be evaluated from baseline to month 6:

- Safety: Type, severity, incidence, and timing of adverse events; incidence of treatment discontinuation due to adverse events; and changes in vital signs, ECG, and laboratory parameters.
- Efficacy: Change in the frequency of generalized tonic–clonic seizures compared to the 6 months prior to the study; EEG: changes in posterior background activity and frequency of epileptiform abnormalities; cognitive function: changes (no worsening or improvement) in neuropsychological assessments; motor function: changes (no worsening or improvement) in motor function assessments; global function and autonomy: changes (no worsening or improvement) in LDS, LESS, ADL, IADL, Barthel Index, Vineland-II, and CGI-I and PGI-CaGI-C scales.
- Biomarkers: Brain FDG-PET: changes (no worsening) in areas of cerebral hypometabolism. High-field brain MRI (3 Tesla): changes (no worsening) in measures of cerebral atrophy. Direct measurement of CSF polyglucosan levels, although potentially informative, is not feasible within the constraints of this pilot study. Thus, brain FDG-PET imaging is included as a surrogate biomarker to assess changes in cerebral metabolism. This choice was primarily due to the significant logistical challenges associated with sample collection and processing for this ultra-rare disease, as well as to the absence of widely available, fully validated assays for the quantitative analysis of CSF polyglucosan levels.

### 3.5. Ethics and Dissemination

#### 3.5.1. Ethics Approval and Consent to Participate

The study protocol has been approved by the Hospital Management Office of the Neurology Unit at IRCCS Casa Sollievo della Sofferenza Hospital, San Giovanni Rotondo (Foggia). Prior to inclusion in the study, written informed consent will be obtained from all patients, who will be informed that participation is completely voluntary and that they can withdraw from the study at any time.

#### 3.5.2. Competing Interest

The authors declare that they have no competing interests. Boehringer Ingelheim, which produces and distributes empagliflozin (Jardiance ^®^) in Italy, provided our hospital with nonfinancial support.

#### 3.5.3. Protocol Amendments

If the protocol requires changing, all revisions and their rationale will be reported by the principal investigator.

#### 3.5.4. Confidentiality

Only the investigators will have access to the data. Authorized personnel will be able to temporarily view the data during the monitoring process.

#### 3.5.5. Access to Data

The datasets utilized and/or analyzed during the study will not be accessible to the public but can be obtained from the corresponding author upon a reasonable request.

#### 3.5.6. Dissemination Policy

The findings from this research will be shared in peer-reviewed journals, presented at both national and international conferences, and distributed to the physicians and participants involved.

### 3.6. In Silico Screening

In silico screening will be performed using molecular docking calculations and reverse ligand-based screening.

For molecular docking calculations, the 3D structures of the targets and SGLT2 inhibitors will be optimized. Specifically, the X-ray structures of hGYS1 and hGLUT1/3 deposited in the Protein Data Bank with PDB codes 7Q13, 5EQG, and 4ZWC, respectively, will be used. Notably, the crystal structure of hGLUT1 presents an inward-open conformation, while that of hGLUT3 presents an outward-open conformation. A homology model will be built from the amino acid sequence (FASTA sequence) of hGLUT1, using the outward-open 3D structure of hGLUT3 (PDB: 4ZWC) as it shares 66% sequence identity. The Schrödinger Suite 2023-4 will be used to generate the structural model using the Prime software. All target structures will then be optimized using the protein preparation wizard (Schrödinger Suite 2023-4) to correct bond orders and add hydrogen atoms and any missing amino acid side chains and cycles. The 3D conformation of empagliflozin and other SGLT2 inhibitors will be processed using LigPrep to generate all possible tautomers and ionization states at a physiological pH of 7.4. Docking simulations will then be performed using Ligand Docking with Energetics (GLIDE) [44,45], which is part of the Schrödinger Suite. Specifically, docking simulations will be performed using the virtual screening workflow implemented in the Schrödinger Suite to consider both open conformations of the GLUT1/3 transporters. For each gliflozin, the docking score and ligand efficiency will be determined relative to the reference ligand for the target considered. To prove the reliability of the docking protocol, redocking simulations will be performed. By redocking the co-crystallized ligands into their respective binding sites and comparing the predicted poses with the experimental ones, using the RMSD (Root Mean Square Deviation) values, the docking methodology will be calibrated, ensuring its accuracy before proceeding with docking calculations with empagliflozin.

As for the reverse ligand-based screening, the protocol implemented in the web platform PLATO (the acronym stands for Polypharmacology pLATform prediction) will be used [34]. As queries, the structure of empagliflozin and other gliflozins (e.g., dapagliflozin) will be used. Target fishing and quantitative bioactivity predictions will be made in PLATO using a multifingerprint similarity algorithm based on ligand-based screening of the ChEMBL database (v.35). The outputs will be examined for possibly identifying further putative protein targets of gliflozins involved in LB formation and neurodegeneration. We can anticipate that, interestingly, among the glifozin targets identified by PLATO, priority is given to glycogen phosphorylase (GP), which should also be implicated in the glycogen hyperphosphorylation and formation of LD bodies.

### 3.7. In Vitro Cell Models Assays

Commercial kits based on a colorimetric cell assay will be used to measure the intracellular glucose and glycogen concentration in neuronal cell lines. This will allow us to evaluate the ability of empagliflozin and other SGLT2 inhibitors to reduce intracellular glucose concentration and glycogen accumulation. The human neuroblastoma cell line SH-SY5Y and the HeK293 cell line will serve as cellular models for these experiments. Glucose and glycogen determination experiments will be performed as previously detailed [46,47,48]. In parallel, cytotoxicity studies will be conducted at the same dosages to assess in vitro the empagliflozin safety profile.

## 4. Expected Results

Many of the therapeutic strategies proposed for the treatment of LD are still under investigation or have shown partial efficacy in patients, failing to halt disease progression. The expected outcomes of this study are to assess the safety and tolerability of empagliflozin in patients with LD and to gather preliminary data on its potential efficacy in improving clinical and neurologic outcomes. We recognize that the lack of a control or comparison group in the proposed single-arm observational design is a limitation. However, to mitigate the absence of a concurrent control group, we will conduct a comparative analysis using data from natural history studies, such as those evaluating metformin or VAL-1221 [9,49]. This approach will allow us to assess potential deviations from the expected disease trajectory and provide a more nuanced interpretation of the observed outcomes. Additionally, the in silico and in vitro studies, complementary and conducted in parallel with the in vivo human study, are expected to provide insights into the mechanisms through which empagliflozin may exert its therapeutic effects in LD. These findings will be important for guiding and supporting future research and development efforts aimed at repurposing empagliflozin or similar drugs for LD. By repurposing an already-approved drug, this research may significantly shorten the time and reduce the costs associated with developing a new therapy for LD patients. This has particular importance considering the rapid progression of the disease and the urgent need for effective treatment options.

The promising preliminary data from this pilot study, if supportive of empagliflozin’s safety and potential efficacy, will lay the groundwork for larger, controlled clinical trials. The specific next steps will include the following: 1. expanding the study cohort by collaborating with other Italian and international centers involved in LD research in order to achieve sufficient statistical power for a robust evaluation of efficacy; 2. implementing a randomized, controlled design, potentially including a placebo arm or comparison to standard of care (subject to ethical feasibility and clinical relevance); 3. further investigating the pharmacological targets through advanced preclinical in vitro and in vivo studies to better understand the mechanism of action; 4. exploring potential biomarkers, including longitudinal MRI and PET assessments, to correlate with clinical outcomes and possibly serve as surrogate endpoints in future trials. The timeline for these studies will depend on the results of this pilot trial, securing necessary funding, and obtaining ethical approval.

## Data Availability

The datasets used and/or analyzed during the study will not be available to the public but will be available from the corresponding author upon reasonable request.

## Abbreviations

The following abbreviations are used in this manuscript:

LD	Lafora disease;
LBs	Lafora bodies;
AILA	Italian Lafora Association;
AMPK	AMP-activated protein kinase;
rhGAA	Recombinant human α-glucosidase;
SGLT2	Sodium-glucose cotransporter 2;
EEG	Electroencephalogram;
GLUT1	Glucose transporter type 1;
GLUT3	Glucose transporter type 3;
GSD	Glycogen storage disorder;
LESS	Lafora Epilepsy Severity Scale;
ADL	Activities of Daily Living;
IADL	Instrumental Activities of Daily Living;
LDS	Lafora Disease Performance Scale.
